# Adjuvant melatonin for uveal melanoma (AMUM): protocol for a randomized open-label phase III study

**DOI:** 10.1186/s13063-023-07245-9

**Published:** 2023-03-26

**Authors:** Ruba Kal Omar, Anna Hagström, Gustav Stålhammar

**Affiliations:** 1 Department of Medicine, Karolinska Institutet, Nobels Väg 6, Stockholm, 171 76 Sweden; 2 Department of Clinical Neuroscience, Division of Eye and Vision, St. Erik Eye Hospital, Karolinska Institutet, Eugeniavägen 12, Stockholm, 171 64 Sweden; 3 St. Erik Eye Hospital, Box 4078, 171 04 Stockholm, Sweden

**Keywords:** Uveal melanoma, Metastases, Melatonin, Randomized open phase III study, Adjuvant treatment

## Abstract

**Background:**

Uveal melanoma is the most common primary intraocular tumor in adults. In Sweden, at least 100 patients are diagnosed with the disease each year. Almost half of the patients develop metastases, with a median survival time of 1 year once metastases are detected. The primary ocular tumor is typically treated with either enucleation or brachytherapy, and no adjuvant treatment is added. Melatonin is an indolamine hormone that has improved survival in previous trials with patients diagnosed with various cancers, including advanced cutaneous melanoma. Side effects have been mild. We aim to investigate if adjuvant treatment with melatonin for 5 years following diagnosis of non-metastasized uveal melanoma can decrease the occurrence of metastases.

**Methods:**

An open-label, prospective, 5-year randomized clinical trial (RCT) will be conducted at St. Erik Eye Hospital. One hundred patients recently diagnosed with non-metastatic uveal melanoma will be randomized to either treatment with adjuvant melatonin 20 mg (4 tablets of 5 mg) at 10 pm for 5 years, or to standard follow-up (control group). The primary outcome measurement is the relative risk for having developed metastases 5 years after randomization. The secondary outcomes are overall survival, risk of developing other cancers, overall survival after detection of metastases, and differences in the occurrence of adverse events (AE) and serious adverse events (SAE) between the groups.

**Discussion:**

Melatonin has been found to positively impact our immune system, inhibit angiogenesis, stimulate apoptosis in malignant cells, and act as a potent antioxidant. Previous clinical trials have used similar doses of melatonin with positive results, particularly in advanced stages of cancer. Previous animal and human studies have found the toxicity of the hormone to be low. Considering the potential benefits and limited risks of melatonin, as well as its global availability, it may be a suitable candidate for an adjuvant treatment in patients with uveal melanoma.

**Trial registration:**

Our trial protocol has been approved and registered by the Swedish Medical Products Agency on June 22, 2022 (EudraCT 2022–500,307-49–00). Our trial registration number is NCT05502900, and the date of registration is August 16, 2022.

## Administrative information

In this trial, 100 subjects with non-metastatic uveal melanoma will be randomized to either treatment with melatonin 20 mg at 10 pm for 5 years or to a control group. The main objective is to investigate whether treatment with melatonin can reduce the risk of developing metastases after 5 years. Only subjects at high risk for metastasis development will be included in the trial. As uveal melanoma has a relatively low incidence rate compared to other cancers, the recruitment period is estimated to take 3 to 4 years.

**Table:** 

EU-examination number	2022–500,307-49–00
Title	Adjuvant Melatonin for Uveal Melanoma: A Randomized Open Phase III Study
Trial code	AMUM
Trial registration	NCT05502900
Protocol version	2.0
Funding	St. Erik Eye Hospital The Swedish Society of Medicine (SLS-971390) The Royal Swedish Academy of Sciences (ME2019-0036) The Swedish Cancer Society (20 0798 Fk) Region Stockholm (RS-2019–1138) The Swedish Eye Foundation (2022–05-02) Karolinska Institutet (FS-2021–0010)
Authors details	R Kal Omar^1^, A Hagström^1^, G Stålhammar^2,3^ ^1^Department of Medicine, Karolinska Institutet, D1:04, 17,176 Stockholm, Sweden ^2^Department of Clinical Neuroscience, Division of Eye and Vision, St: Erik Eye Hospital, Karolinska Institutet, Stockholm, Sweden ^3^St. Erik Eye Hospital, Stockholm, Sweden
Trial sponsor	St. Erik Eye Hospital
Trial period	Q2 2022-Q1 2032
Role of sponsor	The sponsor had no role in study design; collection, management, analysis, or interpretation of data; writing the report; decisions to submit the report for publication; nor did they have ultimate authority over any of these activities

## Introduction

### Background and rationale

About 50% of patients diagnosed with uveal melanoma develop metastases, primarily in the liver [1]. This risk is consistent regardless of which method is chosen to treat the primary tumor [2]. The most common forms of treatment include enucleation (surgical removal of the tumor-containing eye), proton beam irradiation, and plaque brachytherapy—a form of local radiation treatment. Due to very early seeding of micrometastases, the treatment choice typically does not affect the risk of dying from metastatic disease. Micrometastases are clusters of cancerous cells too small to be detected in standard radiological examinations, which migrate from the primary tumor to other organs, such as the liver, where they can remain latent for many years before growing into radiologically detectable tumors, or macrometastases [3]. By the time these metastases are detected, the median survival time is 1 to 2 years [4, 5]. This project’s objective is to investigate if adjuvant treatment with melatonin tablets, following diagnosis of the primary tumor, decreases the risk of developing macrometastases of uveal melanoma.

Previous randomized, placebo-controlled trials investigating various types of solid cancers including cutaneous melanoma have found that melatonin can improve 1-year survival [6]. A similar impact has been seen in a smaller study, where partial responses or stable disease progression was found in 12 of 40 patients (30%) with advanced cutaneous malignant melanoma taking melatonin after an average follow-up of 5 weeks [7]. In a third study, adjuvant treatment with melatonin following surgical resection of lymph node metastasized cutaneous malignant melanoma resulted in significantly fewer patients with progression after an average of 31 months compared to the placebo group [8]. No melatonin-related toxicity was detected in the treatment groups. In a recently published trial with patients with non-small cell lung carcinoma, a significantly improved progression-free survival was seen in a subgroup of patients with advanced-stage disease; however, in another, similar study, no such impact was seen after a year of treatment with melatonin [9, 10]. The overarching factor responsible for melatonin’s impact on cancer is believed to be related to its oncostatic properties, including inhibition of tumor cell proliferation, remodeling of the cytoskeleton, antioxidative effects, and stimulation of the immune system [11–13].

We have recently described the rationale for adjuvant treatment with melatonin for uveal melanoma in a review of the literature [14]. Studies from 1997 and 1998 showed that melatonin can inhibit the growth of uveal melanoma cells with 41 to 50% in a concentration of 0.1 to 10 nM, compared to non-treated cells [15, 16]. Melatonin’s precursors tryptophan and serotonin have not shown a similar effect [16]. Moreover, uveal melanoma could express transmembrane receptors for melatonin. In a study performed by Roberts et al*.*, melatonin and its membrane receptor agonists (MT1 and MT2) were found to inhibit the growth of uveal melanoma cells at low concentrations without impacting non-cancerous melanocytes [17].

In animal studies, the effects of melatonin on uveal melanoma have varied. In a study performed on mice, the cell-line B16-F10 was injected in the tail vein of melatonin knockout mice which were then treated with melatonin 20 mg/kg. After 15 days, there were no significant differences in the number and size of lung metastases compared to controls [18]. Another study performed on a human melanoma xenograft mouse model which was injected with DX3 melanoma cells while receiving injections of melatonin or melatonin analogs UCM 1033 and UCM 1037. When measuring tumor size both 4 weeks after treatment and after euthanization, the injections of UCM 1033 and UCM 1037 were found to inhibit tumor growth by 40 and 90% respectively, while melatonin provided no significant tumor growth suppression [19]. Another study found that mice which received 5 μg/g melatonin in their drinking water daily at the time of melanoma inoculation had a lower size and weight of their melanoma compared to control groups 40 days after administration [20]. In a similar study, athymic mice were injected with melanoma cells and received either 5 μg/g body weight of melatonin in their drinking water together with 0.5% ethanol, 0.5% ethanol alone, or neither melatonin nor ethanol. After 40 days, the mice treated with melatonin had tumors lower in both size and weight [20].

A meta-analysis published in 2005 suggested that melatonin may have similar therapeutic effects in humans. Results found that melatonin treatment in patients with solid tumors was associated with a 44% reduction in the risk of death at 1 year [6]. A clinical trial published in 1991 investigated the effects of melatonin treatment in 40 patients with advanced malignant melanoma, of which, 10 had uveal melanoma [7]. Participants were given oral melatonin in doses ranging from 5 to 700 mg/m^2^ four times a day. After a median follow-up time of 5 weeks, twelve patients showed a response or stable progression of disease. The response appeared to depend, however, on the dose, type of melanoma, and metastasis location with a dose of 75 mg/m^2^ having the best effect with minimal side effects, primarily fatigue [7]. Another clinical trial including five patients with metastatic melanoma studied the effect of adjuvant treatment with 50 mg melatonin taken orally. While four of the five patients had no response to the therapy, one patient continued taking melatonin for over 8 months and had almost total cancer regression [21]. To date, there have been no earlier studies of the long-term effects of melatonin on human patients with uveal melanoma.

## Methods

### Trial design

The study is an open, randomized intervention study without a placebo control. One hundred patients will be randomized to either treatment with melatonin 20 mg at 10 pm, or before bedtime (melatonin group), or to standard follow-up (control group) for a period of 5 years. Aside from treatment with melatonin in the melatonin group, all participants will follow the same schedule for controls and follow-ups as other patients with uveal melanoma. Follow-up examinations are planned 1, 3, 6, and 12 months after primary tumor treatment with either surgery or brachytherapy, followed by annual or semi-annular examinations for life. Radiological examinations, usually with contrast-enhanced ultrasound or computed tomography, are performed every 6 months during the first 5 years. The population treated with melatonin will later be compared to the control group regarding the primary and secondary outcomes.

There are three reasons why we are not using a placebo in this study:

First, our most important outcomes are determined by the results of the radiological examinations (i.e., whether or not metastases are detected), which leaves little room for the placebo effect. Second, the standard clinical routine for uveal melanoma patients is not to use adjuvant treatments after the primary tumor treatment, but instead monitor patients with regular radiological follow-ups. This routine is currently practiced internationally, with little variation between countries. Third, the risk of metastases and survival in the control group are already well known since it has remained consistent over the past several decades with little variation between different populations and time periods [1, 22–35]. It is therefore relatively straightforward to compare our control group to previous cohorts that have not received placebo. Therefore, we will be able to confirm that our control group is representative of bigger populations. This comparison would be more difficult if the control group were to receive a placebo.

### Study objectives and hypothesis

The primary objective of this trial is to investigate whether adjuvant treatment of non-metastatic uveal melanoma with melatonin 20 mg each night reduces the occurrence of metastasis 5 years after randomization (relative risk). The outcome will be measured once the last person in the trial has taken their last melatonin tablet. The secondary objectives for the trial are to examine if adjuvant treatment with melatonin 20 mg each night for 5 years after randomization can:

1. Improve overall survival in 5 years
2. Reduce the hazard of developing another type of cancer in 5 years
3. Improve overall survival after metastasis detection
4. Examine if the occurrence of AE and SAE differs between the melatonin and control groups.

When the last recruited patient has been treated with melatonin for 3 years, interim analyses will be performed. At this point in time, we will compare the occurrence of metastases as well as the overall survival in the melatonin and the control group.

Our hypothesis, based on previous research, is that the group treated with melatonin will have a lower relative risk (RR) for metastasis 5 years after randomization.

### Study setting and recruitment

Participants will be recruited to the trial from St. Erik Eye Hospital in Stockholm as the national management of uveal melanoma patients is centralized to this institution only. The St. Erik Ophthalmic Pathology Laboratory receives most of the eyes with uveal melanoma that have been enucleated throughout the country, meaning that patients will be eligible for the trial regardless of where in Sweden they reside. If the patient is found to meet inclusion and exclusion criteria, they will be contacted via telephone and given information regarding the trial. If the potential participant is interested in taking part in the trial, they will be sent a written consent form and be contacted by a research team member via a service that allows for both video and audio contact, where the potential participant will be able to confirm their identity, ask any questions they may have regarding the trial, and receive additional information. If they wish to participate, they will be asked to sign the consent form (two copies) during the video call and send both of the signed copies in a prepaid envelope to the research team. Once the signed consent form is received by the research team, the participant will be randomized to either the melatonin or the standard treatment group (control group).

As the effectiveness of melatonin will be evaluated in real-life, routine practice conditions, this is considered a pragmatic clinical trial. The study is de-centralized with patients residing in all parts of Sweden eligible, with the drug being delivered to their home address, and with radiological examinations being performed at their home hospitals. Recruitment, randomization, drug prescription, study administration, and follow-up are, however, centralized to St. Erik Eye Hospital.

In contrast to many other western countries, melatonin has not been sold over the counter in Sweden. Since 2020, 3-mg tablets have been sold in packs of 10, at a significant cost to patients. Further, the investigators will be able to detect if trial participants are prescribed melatonin outside the trial through a nationwide digitalized medical journal system.

### Eligibility criteria

#### Inclusion criteria

1. Participants must be 18 years or older.
2. Participants must give written consent for participation in the trial.
3. Participants must be clinically or histopathologically diagnosed with malignant melanoma originating from the choroid and/or the ciliary body.
4. If the patient has already received treatment with melatonin for another reason, they have had a wash-out period where no melatonin was taken for a least 2 weeks prior to randomization.

In addition to meeting these three criteria, at least one of the following criteria (5–11) must be met as well:

5. Participant’s tumor is categorized as a size of T3d or higher, corresponding to stage IIIB or higher (according to AJCC TNM version 8) [36].
6. Participant’s tumor is of a size category “large” according to modified COMS criteria (largest basal diameter > 16 mm or thickness > 8 mm) [22].
7. Participant’s tumor is of a size category of T2a or higher prior to brachytherapy and has since recurred after brachytherapy [36].
8. Participant’s tumor is composed of an epithelial cell type (> 5 epithelial cells/hpf and > 90% epithelial cells in total).
9. Participant’s tumor has a low immunohistochemical expression of the BAP-1 protein [37–41].
10. Participant’s tumor has 9 or more mitoses per 40 hpf.
11. Participant has via another published and validated prognostic test been found to have a risk of 60% or higher for developing metastases after 5 years, for example, “Gene expression class 2,” “TCGA group D,” measurements of protein levels in serum, or similar [3, 42, 43].

#### Exclusion criteria

1. Participant is oversensitive to melatonin or any of the tablets’ contents.
2. Participant has metastases detectable with radiological examinations or other methods at the time of recruitment (note that the development of metastases later on during the course of the trial does not disqualify a participant).
3. Participant is unable to provide consent.
4. Participant has compromised liver function, for example due to liver cirrhosis or hepatitis.
5. Participant is pregnant, or a woman of child-bearing potential, WOCBP. Any woman between menarche and menopause is considered to have child-bearing potential, if not permanently sterile through hysterectomy, bilateral salpingectomy, or bilateral oophorectomy. Menopause is defined as absence of menstruation for ≥12 months without other explanation.
6. Participant is breastfeeding or wishes to breastfeed before the planned completion of melatonin treatment. Women admitted to the trial who begin breastfeeding during the course of the trial before the studies completion will be withdrawn from the study.
7. Participant has epilepsy.
8. Participant is treated, for more than 4 weeks, with CYP1A2-inhibitors fluvoxamine, quinolones, verapamil, or cimetidine; with combined hormonal contraception (containing ethinyl estradiol and progestogen); with hormonal substitution therapy; with 5-methoxypsoralen or 8-methoxypsoralen; or with cimetidine. If the participant, after being included in the trial, starts a lasting treatment (more than 4 weeks) with one of the previously mentioned substances, the participant does not need to cease participation in the trial but instead can refrain from taking melatonin during their treatment with these substances and then resume their melatonin treatment with the ordinary, daily dose of 20 mg at 10 pm.
9. Participant was diagnosed with primary uveal melanoma more than 12 months prior to the assessment of eligibility.

### Informed consent

A research member will obtain informed consent from the trial participants. In Sweden, informed consent must be collected by a licensed physician. The informed consent form will be sent to the participant via post which the participant will sign if they wish to participate in the trial and send back to the research team who will also sign it and send a copy back to the participant. The participant will then be randomized to either the melatonin group or the control group. Participants will be informed about their right to withdraw from the trial at any time without a need for an explanation. Participants will be asked to consent to monitors, inspectors, research members, and regulatory authorities having access to their data that is relevant to the trial. This trial does not involve collecting biological specimens for storage.

### Interventions

#### Randomization and allocation

The randomization takes place when the participant has sent their signed informed consent form to the research team, agreeing to be a part of this clinical trial. Fifty sheets of paper labeled “melatonin” and 50 sheets of paper labeled “control” will be preserved in a total of 100 sealed, identical envelopes, mixed in one box. When written informed consent is received from a participant, we will randomly choose one of these envelopes and open it. The content will then determine the randomization of the patient, i.e., melatonin or control. When the process of randomization is complete, the participant will be informed of the outcome. If they have been randomized to the melatonin group, they will receive a prescription for melatonin and an order will be placed for 15 containers of melatonin tablets (corresponding to 1 year of consumption) each year during the 5-year period. The participating research staff as well as the participants will therefore be aware of their randomization outcome, either to treatment with melatonin or to composing the control group.

#### Blinding

This trial is not blinded. The study design for this clinical trial is open-label, where blinding will only occur during data analysis by outcome assessors. Therefore, a procedure for unblinding is not necessary.

#### Intervention

**Fig. 1:**
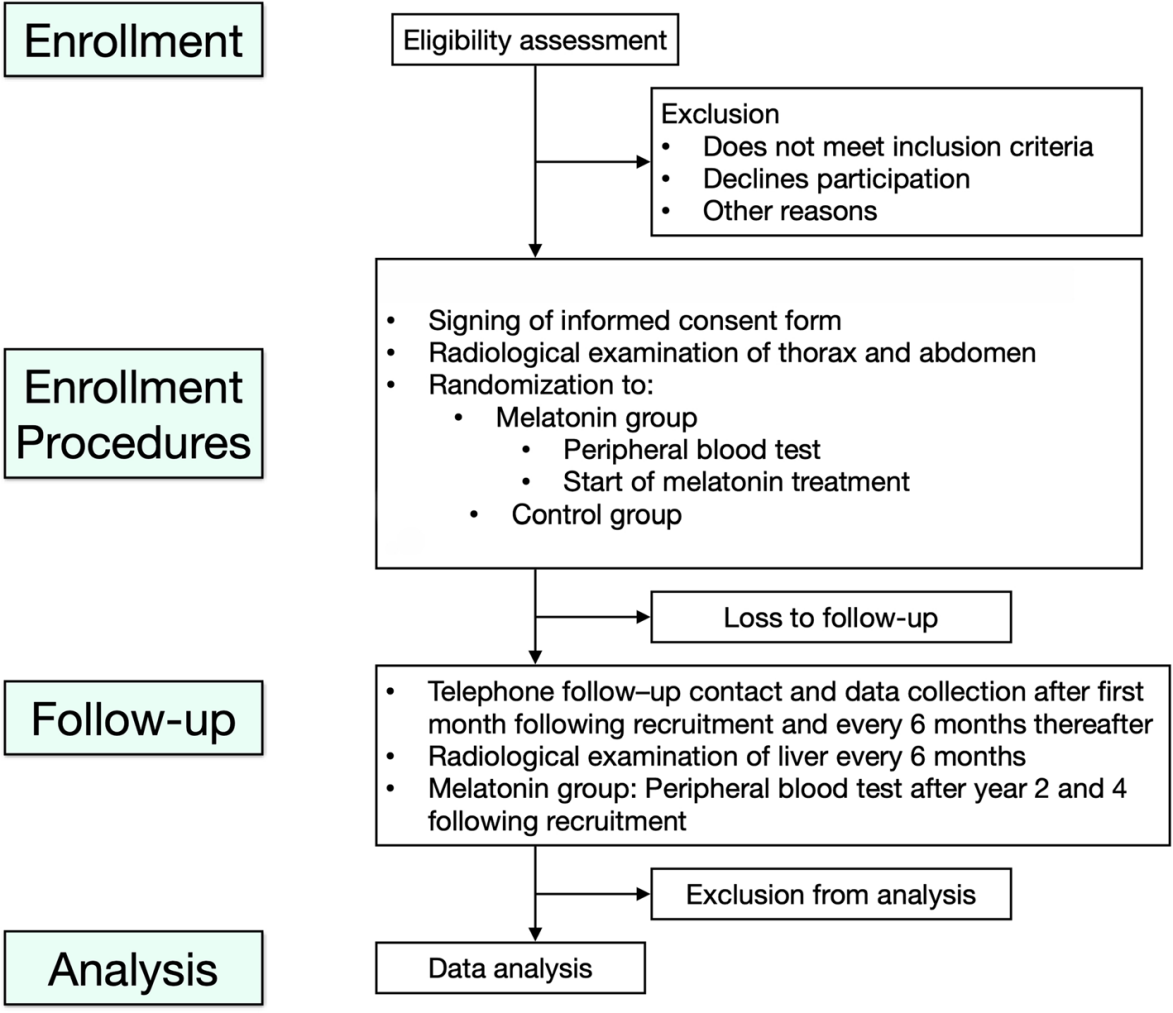
CONSORT diagram describing study design

Participants will be equally randomized into two groups: the melatonin group (treated with 20-mg melatonin each night for 5 years) or the control group (no intervention). When a patient is recruited and randomized to the melatonin group, blood samples will be ordered by a licensed physician regarding creatinine, electrolytes (sodium, potassium), and alanine transaminase (ALAT). This blood test will be repeated after 2 and 4 years (Fig. 1 and Tables 1 and 2).

**Table 1:** Study design

Time	Event in clinical routine	Other events for trial subjects
At diagnosis	Clinical examination of the eye. Radiological examination of the thorax and abdomen. Primary treatment^a^	Provide information about the trial. Screen participant for inclusion and exclusion criteria Obtain written consent. Give medication instructions. Order blood tests to measure kidney function, electrolytes, and liver function. Prescribe melatonin 20 mg every night for 5 years
1 month	A	Ask about AE and SAE, and about prescription compliance
6 months	B	Ask about AE and SAE, and about prescription compliance
1 year	A + B	Ask about AE and SAE, and about prescription compliance
1.5 years	B	Ask about AE and SAE, and about prescription compliance
2 years	A + B	Ask about AE and SAE, and about prescription compliance. Ordering blood tests to measure kidney function, electrolytes, and liver function
2.5 years	B	Ask about AE and SAE, and about prescription compliance
3 years	A + B	Ask about AE and SAE, and about prescription compliance
3.5 years	B	Ask about AE and SAE, and about prescription compliance
4 years	A + B	Ask about AE and SAE, and about prescription compliance. Order blood tests to measure kidney function, electrolytes, and liver function
4.5 year	B	Ask about AE and SAE, and about prescription compliance
5 years	A + B	End of melatonin treatment. Evaluation of overall survival, metastasis-free survival, and proportion of subjects with metastases^b^. Ask about AE and SAE

A = Follow-ups at St. Erik Eye Hospital or at the participant’s local eye clinic. Examination of the eye, for subjects treated with radiation, or of the prosthesis and/or orbit, for subjects treated with enucleation. Follow-up interval ± 4 weeks during the first year and ± 8 weeks after the first year

B = Radiological examination of the liver with ultrasound or computer tomography every 6 months for the first 5 years after diagnosis; can be performed at the participant’s local healthcare clinic. Follow-up interval ± 4 weeks during the first year and ± 8 weeks after the first year

^a^Primary treatment of uveal melanoma in the eye is either enucleation (surgical removal of the eye) or brachytherapy (local radiation therapy of the eye)

^b^Evaluation of the proportion of subjects with metastases is performed through data collection via registries, chart documentation, and statistical work. It therefore does not require subjects’ active participation

**Table 2:** Flowchart

**Time**	**Screening** − 30 days before 1 m visit	**Month 1** ± 7 days	**Month 6** ± 4 weeks	**Year 1** ± 4 weeks	**Year 1.5** ± 8 weeks	**Year 2** ± 8 weeks	**Year 2.5** ± 8 weeks	**Year 3** ± 8 weeks	**Year 3.5** ± 8 weeks	**Year 4** ± 8 weeks	**Year 4.5** ± 8 weeks	**Year 5** ± 8 weeks
Inclusion/exclusion	x											
Informed consent	x											
Randomization melatonin/control group	x											
Medical history, current medication	x											
Medication instruction	x											
Radiological examination—liver			x	x	x	x	x	x	x	x	x	x
Radiological examination—thorax and abdomen	x											
Blood samples	x					x				x		
AE and SAE, prescription compliance		x	x	x	x	x	x	x	x	x	x	x
End of study												x

### Data collection

A case report form (CRF) will be established in paper form and used for data collection, where subjects will be identified through a trial-specific number only. The examiner is responsible for assuring that registered information is correct and complete and that the reporting is performed according to the predetermined timeline. The principal investigator will sign the completed CRF and a copy will be archived where the trial takes place.

### Follow-up

The subjects will take their medication (tablets) at home and confirm that they have received each package of melatonin sent to them. They will, 1 month following recruitment, be contacted via telephone and asked about their compliance to the treatment. The participants will then continue to be contacted via telephone every 6 months throughout the trial period and asked questions about compliance, adverse events (AE), and serious adverse events (SAE). After the termination of the trial, the subjects will continue their yearly control visits at St. Erik Eye Hospital, or at their local ophthalmology clinic, for the remainder of their lives, as all uveal melanoma patients do.

### In the event of detection of metastases

If a suspected metastasis is detected in radiological examinations and then verified with a biopsy, the patient will be referred by their physician to an oncology clinic in their home region. This is a standard clinical routine and will not be impacted by this trial. The oncology clinic will make a decision regarding next steps to investigate, examine, and treat the metastases. Patients with metastases typically receive systemic chemotherapy, immunotherapy with so-called checkpoint inhibitors, and/or regional perfusion treatment of the liver where high doses of chemotherapy are injected into the liver’s circulatory system. These treatments are demanding for patients and can result in side effects such as fatigue, susceptibility to infection, dry mucous membranes, and pain. The average survival following metastasis detection is about 1 year. Metastasis detection does not alter the melatonin doses or treatment instructions in this trial. Melatonin given at a dose of 20 mg at 10 pm has shown particularly positive effects in advanced stages of cancer in earlier studies [10].

It has also been reported that systemic chemotherapy is more easily tolerated in patients who are simultaneously treated with melatonin, particularly in regard to neurotoxicity, thrombocytopenia, weight loss, and asthenia [44]. Furthermore, there are fewer cases of alopecia (hair loss) and infection in patients taking 20 mg of melatonin at 10 pm while undergoing radiotherapy [45]. If participants in this trial take melatonin from the time that their metastases are detected, the metastases will be included in both the intention-to-treat and the per-protocol analysis. Survival in the melatonin group following metastasis detection will therefore be counted in the patients still taking melatonin.

### Withdrawal

Subjects may voluntarily withdraw from the trial at any time without impacting further standard treatment. The principal investigator reserves the final right to terminate the trial for a specific subject due to unacceptable side effects or poor compliance. If a subject terminates their participation in this trial, their trial-specific ID will not be re-used. They will not be able to re-join the trial. No further data will be collected; however, data that has already been collected will be used for the final analysis of the trial. Participants who withdraw within 6 months following randomization will be replaced by newly recruited and randomized patients with a ratio of 1:1, i.e., if two participants withdraw from the trial within 6 months, they will be replaced by two new participants. Patients who withdraw from the trial after 6 months following randomization will not be replaced.

### Sample size

Our power calculations will be based on the following:

1. A previously calculated metastasis incidence of 60% 5 years following diagnosis for individuals not taking melatonin (participants in the control group). This is a relevant calculation considering the high-risk population recruited based on our inclusion criteria [46–49].
2. An earlier study which included study participants with surgically removed lymph node metastases of malignant melanoma which showed that 31% (5 of 16 participants) of those treated with 20 mg of evening melatonin developed distant metastases compared to 71% (10 of 14 participants) of those not treated with melatonin [8].

Based on this previous research, the following assumptions are made:

1. The number of participants in the melatonin group which will have developed distant metastases after 5 years: 30%
2. The number of participants in the control group which will have developed distant metastases after 5 years: 60%.
3. Alpha: 0.05
4. Beta: 0.2
5. Power: 80%
6. Results: 42 participants must be included in each group, i.e., both the melatonin and the control group, in order to achieve statistical significance (Table 3). To obtain a safety margin in case some participants withdraw from the trial, we will strive after a group population of 50 participants per group.

**Table 3:** Power calculation

**Melatonin, *n* = **	42
**Control, ***n*** = **	42
**Incidence, melatonin group**	30%
**Incidence, control group**	60%
**Alpha**	0.05
**Beta**	0.2
**Power**	0.8

### Outcomes

The primary outcome is the number of patients which develop metastasis in the melatonin group vs. the control group. This will be evaluated as a relative risk (RR), with a 95% confidence interval. The secondary outcomes include the following:

1. Overall survival 5 years from randomization in the melatonin group vs. control group, evaluated with Kaplan-Meier curves and the Log-rank test.
2. Overall survival after detection of metastases in the melatonin group vs. control group, was evaluated with Kaplan-Meier curves and the Log-rank test.
3. Hazard for having developed other cancers within 5 years from randomization in the melatonin group vs. control group, evaluated as Cox regression hazard ratio (HR).
4. Number of patients with AE and SAE in the melatonin group vs. control group, assessed by CTCAE and evaluated as relative risk (RR) with a 95% confidence interval.

Other outcome measures:

1. Interim analysis: number of patients developing metastasis within 3 years in the melatonin group vs. control group, evaluated as relative risk (RR), with 95% confidence interval.
2. Interim analysis: overall survival in 3 years in the melatonin group vs. control group, evaluated with Kaplan-Meier curves and the Log-rank test.

#### Clinical effects

Our primary and secondary outcomes for the clinical effects of melatonin treatment will be performed through data collected from patient charts, where it is registered if the subject has developed metastasis, if they are deceased, and/or if they have received another cancer diagnosis in addition to uveal melanoma.

#### Clinical safety

Our outcomes for clinical safety will be the number of adverse events (AE), the number of serious adverse events (SAE), and the number of suspected unexpected serious adverse reactions (SUSAR) in the melatonin group and control group respectively. These will be measured through data collected from CRF forms and patients’ chart documentations. The subjects are encouraged to contact us if they experience side effects, or new or worsened symptoms. They will be asked about AE and SAE at 3 months and 1 year after starting melatonin treatment, and thereafter every 6 months until 5 years have passed since recruitment.

### End of study definition

The trial will be terminated when the last participant in this trial has had their 5-year control (Last Subject Last Visit (LSLV)). No further treatment or contact with the participants will be made after this point of time.

### Statistical analysis

We will be analyzing our data according to “intention to treat” where all participants randomized to either the melatonin or control group are included in the said group even if some terminate their participation in the trial or are excluded from the trial later on. We will at the same time analyze our data “per protocol” where the participants who terminate their participation during the trial or are noncompliant to melatonin treatment are excluded from the analysis.

To answer our primary and secondary objectives, we will use both the chi-square test and the Log-rank test of cumulative survival with associated 95% confidence intervals and *p*-values. Relative risk will be calculated as the probability of metastasis in the melatonin group divided by the risk of metastasis in the control group.

- *P* values less than 0.05 will be considered significant, and all tests will be double-tailed, or two-sided.
- The Shapiro–Wilk test will be used to confirm if our continuous data is normally distributed.
- A Student’s *t*-test will be used to compare the differences in normally distributed continuous data within the melatonin and control groups, respectively. Data found not to be normally distributed data will be compared using the Mann–Whitney *U*-test.
- In order to compare the risk of death and metastasis between the melatonin and control group, we will also use a Cox Regression, adjusted for covariates such as tumor size, T category, cell type, presence of extra scleral growth, presence of extracellular matrix patterns, and BAP-1 expression.

### Interim analyses

An interim analysis will take place 3 year after randomization of the last patient in the trial.

## Oversight and monitoring

### Data monitoring

To ensure that the trial is performed according to the protocol—that the data is collected, documented, and reported according to ICH-GCP as well as regarding ethical and regulatory requirements—the trial will be monitored by an independent monitor before the start of the trial, during the course of the trial, and after the trial ends, through Karolinska Trial Alliance. This will take place according to the monitoring plan for the trial and aims to ensure the rights, safety, and well-being of participants as well as to oversee that data in the CRF forms is filled in correctly.

### Harm

#### Registration of events

An AE is defined as any unfavorable event or worsening of an existing medical state in a subject that receives a trial medication, regardless of causation. An SAE is a serious event where a specific dose leads to death, is life-threatening, requires health care, causes remaining or massive invalidation or disability, or causes an abnormality or malformation. A SUSAR is defined as a side effect or an incident that is serious, unexpected, and suspected to be caused by the treatment. Subjects will be encouraged to contact the principal investigator if any of these incidents occur. They will also be specifically asked about AE, SAE, and SUSAR after 1 month of treatment and at regular follow-ups throughout the course of the trial. All AE will be registered in the CRF and evaluated regarding causation, intensity, and if the incident is classified as an AE or an SAE. SAE will be reported in a specific form and submitted to the principal investigator within a day. SUSAR will be evaluated by a licensed physician and further reported to The Swedish Medical Products Agency and the physicians that have met the subject during check-ups. Lethal or life-threatening SUSAR will be reported within a week and complemented with other relevant information within the 8 days following, while other types of SUSAR will be reported within 15 days.

#### Evaluation of events

The principal investigator will evaluate the possible causation between AE/SAE and melatonin usage. If such an association is found, the subjects will be followed until declared healthy, until a “near healthy” status is declared, or until the principal investigator considers no further need for follow-up. All AE/SAE will be categorized as probably related, possibly related, or not related. Probably related events are defined as clinical events, including abnormal laboratory analysis results occurring at a reasonable time following melatonin administration, if the event is unlikely to be related to an underlying disease or another medication, or if the symptoms are previously known side effects of melatonin. Possibly related events are defined as clinical events, including abnormal laboratory analysis results occurring after a reasonable time following melatonin treatment, where the event can be explained by the usage of melatonin, but there is not sufficient information to ensure the causation exists. Not related events are defined as clinical events including abnormal laboratory analysis results occurring at an unreasonable time as related to melatonin treatment, thereby suggesting the event is likely not related to the intervention (melatonin) and can be explained by another medication or underlying disease.

Unfavorable medical events will be classified and graded according to the Common Terminology Criteria for Adverse Events (CTCAE), version 5.0. based on the following:

**Table:** 

Grade 1:	Mild Asymptomatic or mild symptoms Only clinical or diagnostic observations; intervention not indicated
Grade 2:	Moderate Minimal, local, or non-invasive interventions indicated; affects age-adjusted ADL
Grade 3:	Severe Medically significant but not acutely life-threatening; hospitalization or lengthened health care indicated; disabling; limits personal ADL
Grade 4:	Life-threatening Life-threatening consequences, acute interventions indicated
Grade 5:	Death related to side effects

In urgent situations, the principal investigator will immediately take urgent safety measurements required to protect the subjects, such as temporarily terminating the clinical trial medication or implementing additional monitoring measures. An overdose of medication associated with AE will be registered as a diagnosis or symptom in the CRF, and in the subject’s journal documentation if no associated symptoms are found. Women of fertile age will be excluded from this trial.

All study participants will be insured via LÖF (the Swedish patient insurance).

### Management and auditing

The project management group for this trial will meet once or twice monthly to review trial conduct. This group is composed of a primary investigator and two research team members who will be providing day-to-day support to the trial. All group members will share responsibility for running the trial, with tasks such as recruiting and randomizing patients, conducting follow-ups, collecting data, and answering participants’ questions. They will communicate regularly via email and telephone in addition to the monthly meetings. No trial steering group is assigned for this trial; however, data is monitored by Karolinska Trial Alliance before launching the trial, at the beginning of the trial, after 1 month, after 3 months, and then every 6 months during the trial. The Swedish Medical Products Agency will review trial safety data and adherence to regulations once a year.

#### Dissemination plans

This study protocol follows the Standard Protocol Items: Recommendations for Interventional Trials (SPIRIT) guidelines [50]. In order to communicate the trial results to participants, healthcare professionals, and the public, we plan to publish our findings in peer-reviewed journals and present them at both domestic and international scientific conferences.

## Discussion

Melatonin is a hormone which positively impacts our immune system, inhibits angiogenesis, and stimulates apoptosis in malignant cells. This hormone may be a suitable candidate for an adjuvant treatment in uveal melanoma, particularly for patients with high risk for metastasis development but who do not yet have detectable metastases at diagnosis [14].

A dose of 20 mg per day or higher has been used in at least 14 earlier randomized clinical studies for patients that have been diagnosed with various forms of cancer [8–10, 13, 16–18, 21, 30, 44–53]. In eight of these studies, the treatment has been used for at least a year in a total of 651 patients (30 + 356 + 49 + 14 + 12 + 55 + 35 + 100) in the melatonin group [8–10, 13, 44, 45, 49, 51, 52]. In these clinical trials when the median patient had been treated with 20 mg melatonin per day for a year or longer, no blood samples or other tests were taken regarding subclinical AE, i.e., leucopenia, thrombocytopenia, hyperglycemia, hypokalemia, hyponatremia, hyperbilirubinemia, and proteinuria [8–10, 13, 44, 45]. One exception is the clinical trial performed by Lissoni et al. where 100 patients with metastasized lung cancer were randomized to either systemic chemotherapy or systemic chemotherapy with melatonin. In this study, thrombocytopenia was evaluated, which was significantly less frequent in the melatonin group [44]. In another study, blood samples were taken to measure white blood cells and eosinophils during each cycle of IL-2 in patients with advanced gastrointestinal cancer treated with 50 mg of melatonin at 10 pm during 1 week per month as an adjuvant treatment [51].

Regarding toxicity and adverse events, these studies have in general reported few side effects of melatonin, such as mild anxiety, though subjects in another study experience decreased anxiety [8, 45]. In most studies, no differences in side effect outcomes between melatonin and placebo groups were found regarding melatonin-related toxicity including nausea or other gastrointestinal issues [8]. One study found no significant differences in Quality OF Life (QOL) QLQ-C30 or in side effect subcategories including physical, emotional, cognitive, social, fatigue, nausea, pain, insomnia, lack of appetite, obstipation, or diarrhea [9]. No cardiovascular, pulmonal, renal, or hematological complications were observed when using melatonin as an adjuvant treatment to IL-2 in patients with advanced gastrointestinal cancer [51]. To the contrary, some studies reported a better cytotoxic chemotherapy response regarding neurotoxicity, thrombocytopenia, and asthenia in groups receiving melatonin as well as significantly fewer complications involving infections related to radiation therapy and/or steroid treatments [44, 45]. Other studies found an improvement in the Karnofsky Performance Scale in the melatonin group [51, 52].

The toxicity of melatonin has been low in earlier animal and human studies. In animals, an LD50 (lethal dose in 50% of experimental animals) could not be established since no doses, to date, have been proven to be lethal. The highest used daily dose known for the sponsor is 800 mg/kg body weight in mice (corresponding to 60 g for a 75-kg body weight). A higher dose could not be attained since higher doses were not water-soluble [53]. In one study, 5 patients with hyperpigmented skin who received 1 g melatonin/day for 25–30 days were observed to have slightly lower levels of growth hormone, luteinizing hormone, follicle-stimulating hormone, and cortisol during treatment. However, there were no changes in their hematological status (white blood cells, hemoglobin, hematocrit, thrombocytes, reticulocytes, and differential white blood cell count), plasma creatinine, uric acid, calcium, phosphate, transaminases, LDH, bilirubin, total protein, albumin, thyroxin, or retinal appearance [54]. In a study performed on 11 patients with Parkinson’s disease that received 50–100-mg melatonin daily, no changes in serotonin levels in urine were observed. At least one other previous study has had participants take 20 mg of melatonin every evening for 5 years [55]. In this study, patients with metastasized non-small cell lung cancer were randomized to treatment with either cisplatin and etoposide or to cisplatin, etoposide, and melatonin. The patients who received melatonin were found to have both significantly better overall survival and significantly fewer reported side effects [55].

Animal studies indicate that melatonin can downregulate the pituitary–gonadal axis, which may result in hypogonadism or delayed puberty [54, 56]. Treatment with low doses (2 mg) of melatonin in men did not, however, change blood levels of testosterone or luteinizing hormone [57]. Treatment with melatonin in women has been shown to lower the secretion of gonadotropin in a rapport from a clinical phase II study where 1400 women were treated with 75-mg melatonin each evening for 4 years, though no other side effects were observed [58]. Considering these findings, prepubescent children and women of reproductive age will be excluded from this trial.

Even if melatonin has shown potential as an adjuvant treatment of cancer including uveal melanoma, the timing of melatonin consumption is relevant as it is a hormone which is naturally secreted at 10 pm in the absence of light [59]. Melatonin given at the wrong time point in the circadian rhythm might have the opposite effect [14]. Melatonin injections given in the morning have been shown to stimulate the growth of fibrosarcoma and Erlich tumors, while injections at 10 pm inhibit tumor growth [60]. Furthermore, melatonin distribution on multiple occasions during both day and night was found to exacerbate depressive symptoms [61]. In a study performed on rats, treatment with peritoneal injections of 100 μg melatonin/day (corresponding to 30 mg/day given to an adult) was found to have more photoreceptor damage in the retina after illumination with high-intensity fluorescence light of 1600 lx for 24 h, compared to animals treated with placebo [62]. Therefore, as melatonin is a hormone important to our natural circadian rhythm, it is important to consider the timing of melatonin consumption, ideally at 10 pm.

If melatonin is found to prevent the development of metastases, there would be benefits for both the trial participants who took melatonin during the trial and for patients diagnosed with uveal melanoma in the future. By sharing our results in research articles or by other means, this benefit could become available to patients beyond Sweden’s borders as well. Melatonin is relatively accessible worldwide and could potentially reduce mortality for a large proportion of the 7000 patients diagnosed with the disease annually around the globe. We therefore believe that the benefits of this trial outweigh the limited potential risks.

Since data regarding the effects of melatonin as an adjuvant treatment in uveal melanoma is limited, this clinical trial will be performed to investigate if adjuvant treatment with melatonin following diagnosis of the primary tumor decreases the risk of developing macrometastases.

## Trial status

The recruitment of patients for this trial will begin on September 23, 2022. The authors expect to complete this procedure by September 2026. Our trial protocol has been approved and registered by the Swedish Medical Products Agency on June 22, 2022 (EU No. 2022–500,307-49–00). Our trial registration number is NCT05502900.

## Protocol amendments

Crucial changes in the signed protocol will be registered in the protocol amendment and further submitted through CTIS for approval.

## Data Availability

All investigators as well as the monitoring organization will have access to the final trial dataset. There are no contractual agreements that limit such access for investigators. The collection of personal information including but not limited to names, addresses, social security numbers, and other contact details about the trial’s participants, and disclosure of such information to any party, is regulated by The Swedish Medical Products Agency and subject to approval from the Swedish Ethical Review Authority.

## Abbreviations

AE: Adverse event
CRF: Case report form
CTIS: Clinical trial information system
CTR: Clinical trial regulation
GCP: Good clinical practice
ICH: International Council for Harmonization
ITT: Intention-to-treat
PP: Per-protocol analysis
SAE: Serious adverse event/serious adverse reaction
SUSAR: Suspected unexpected serious adverse reaction
